# Infection risk reduction with povidone-iodine rectal disinfection prior to transrectal prostate biopsy: an updated systematic review and meta-analysis

**DOI:** 10.1007/s00345-024-04941-2

**Published:** 2024-04-23

**Authors:** Ichiro Tsuboi, Akihiro Matsukawa, Mehdi Kardoust Parizi, Jakob Klemm, Stefano Mancon, Sever Chiujdea, Tamás Fazekas, Ekaterina Laukhtina, Tatsushi Kawada, Satoshi Katayama, Takehiro Iwata, Kensuke Bekku, Koichiro Wada, Motoo Araki, Shahrokh F. Shariat

**Affiliations:** 1 Department of Urology, Comprehensive Cancer Center, Medical University of Vienna, Vienna General Hospital, Währinger Gürtel 18-20, 1090 Vienna, Austria; 2 Department of Urology, Shimane University Faculty of Medicine, Shimane, Japan; 3 Department of Urology, Okayama University Graduate School of Medicine, Dentistry and Pharmaceutical Sciences, Okayama, Japan; 4 Department of Urology, Jikei University School of Medicine, Tokyo, Japan; 5 Department of Urology, Shariati Hospital, Tehran University of Medical Sciences, Tehran, Iran; 6 Department of Urology, University Medical Center Hamburg-Eppendorf, Hamburg, Germany; 7 Department of Biomedical Sciences, Humanitas University, Pieve Emanuele, Italy; 8 Department of Urology, Spitalul Clinic Judetean Murures, University of Medicine, Pharmacy, Science, and Technology of Targu Mures, Mures, Romania; 9 Department of Urology, Semmelweis University, Budapest, Hungary; 10 Institute for Urology and Reproductive Health, Sechenov University, Moscow, Russia; 11 Department of Urology, University of Texas Southwestern, Dallas, TX USA; 12 Department of Urology, Second Faculty of Medicine, Charles University, Prague, Czech Republic; 13 Hourani Center for Applied Scientific Research, Al-Ahliyya Amman University, Amman, Jordan; 14 Karl Landsteiner Institute of Urology and Andrology, Vienna, Austria; 15 Research Center of Evidence Medicine, Urology Department Tabriz University of Medical Sciences, Tabriz, Iran; 16 Department of Urology, Weill Cornell Medical College, New York, NY USA

**Keywords:** Antibiotic prophylaxis, Povidone-iodine, Transrectal ultrasound-guided prostate biopsy

## Abstract

**Background:**

To prevent infectious complications after transrectal ultrasound-guided prostate biopsy (TRUS-PB), some studies have investigated the efficacy of rectal disinfection using povidone-iodine (PI) and antibiotic prophylaxis (AP).

**Objective:**

To summarize available data and compare the efficacy of rectal disinfection using PI with non-PI methods prior to TRUS-PB.

**Evidence acquisition:**

Three databases were queried through November 2023 for randomized controlled trials (RCTs) analyzing patients who underwent TRUS-PB. We compared the effectiveness of rectal disinfection between PI groups and non-PI groups with or without AP. The primary outcomes of interest were the rates of overall infectious complications, fever, and sepsis. Subgroups analyses were conducted to assess the differential outcomes in patients using fluoroquinolone groups compared to those using other antibiotics groups.

**Evidence synthesis:**

We included ten RCTs in the meta-analyses. The overall rates of infectious complications were significantly lower when rectal disinfection with PI was performed (RR 0.56, 95% CI 0.42–0.74, *p* < 0.001). Compared to AP monotherapy, the combination of AP and PI was associated with significantly lower risk of infectious complications (RR 0.54, 95% CI 0.40–0.73, *p* < 0.001) and fever (RR 0.47, 95% CI 0.30–0.75, *p* = 0.001), but not with sepsis (RR 0.49, 95% CI 0.23–1.04, *p* = 0.06). The use of fluoroquinolone antibiotics was associated with a lower risk of infectious complications and fever compared to non-FQ antibiotics.

**Conclusion:**

Rectal disinfection with PI significantly reduces the rates of infectious complications and fever in patients undergoing TRUS-PB. However, this approach does not show a significant impact on reducing the rate of sepsis following the procedure.

**Supplementary Information:**

The online version contains supplementary material available at 10.1007/s00345-024-04941-2.

## Introduction

Transrectal ultrasound-guided prostate biopsy (TRUS-PB) and transperineal ultrasound-guided prostate biopsy (TPUS-PB) have been the main procedures to diagnose prostate cancer [1]. Despite the recommendations of the European Association of Urology (EAU) guidelines favoring TPUS-PB over TRUS-Bx, the transrectal approach is still widely utilized worldwide, mainly due to the elaborate technical requirements of TPUS-Bx. However, TRUS-Bx leads to higher incidence of infectious complications based on the translocation of rectal bacteria during the procedure. The estimated incidence rate of infectious complications by TRUS-PB, such as acute bacterial prostatitis, fever, and sepsis, is reported to be as high as 6.3% [2, 3].

Several studies, including randomized controlled trials (RCT), have been conducted to assess the efficacy of PI disinfection of the rectum in reducing infectious complications after TRUS-PB [2, 4–9]. These studies have indicated that PI disinfection can reduce infectious complications, leading to the EAU guidelines to recommend rectal disinfection with PI prior to TRUS-PB [10]. Although previous systematic reviews and meta-analyses compared the efficacy of PI to reduce infection between the PI group and non-PI group and between the PI plus AP group and AP monotherapy group, the efficacy of using PI in reducing sepsis remains uncertain when comparing PI plus AP and AP monotherapy [4, 9].

Therefore, the aim of this systematic review and meta-analysis was to evaluate the efficacy of pre-TRUS-PB disinfection with PI plus AP compared to AP monotherapy. We aimed to reassess the role of PI in mitigating infectious complications. Furthermore, we make efforts to clarify and verify the effectiveness of PI in reducing the incidence of sepsis.

## Evidence acquisition

We registered the study with the International Prospective Register of Systematic Reviews (PROSPERO: registration number: CRD42023476473). This systematic review and meta-analysis was conducted in line with the Preferred Reporting Items for Systematic Reviews and Meta-analyses (PRISMA) statement (PRISMA 2020 checklist, Supplementary Table 1).

### Search strategy

On November 1st, 2023, the PubMed, Scopus, and Web of Science databases were searched to identify studies investigating the effectiveness of disinfection with PI before TRUS-PB. The search terms included: “prostate biopsy”, “povidone iodine”. The detailed search strategy is shown in Supplementary Appendix 1. Two investigators independently performed an initial screening based on the titles and abstracts and noted the cause of exclusion of ineligible reports. Full texts were retrieved and evaluated for eligibility. In addition, hand searches of reference lists were performed to identify additional studies of interest. In the case of discrepancies, the disagreements were solved by consensus among the authors.

### Inclusion and exclusion criteria

We included studies that analyzed patients, who underwent TRUS-PB with rectal disinfection using PI with or without AP and compared them with patients who underwent TRUS-PB without PI disinfection, to assess the incidence of pooled infectious complications, including fever, sepsis, symptomatic urinary tract infection (UTI) only in RCTs. Analyses of different subgroups were performed to evaluate the varying results among patients treated with fluoroquinolones vs. those treated with other types of antibiotics. Reviews, meta-analyses, letters, editorials, meeting abstracts, authors’ replies, case reports, and non-English articles were excluded. In the case of duplicate publications, either the higher quality or the most recent publication was selected. We scanned references of included manuscripts for additional studies of interest.

### Data extraction

Two reviewers separately extracted data on baseline study and patient’s characteristics. The first author’s name, published year, inclusion criteria, exclusion criteria, type of prophylactic antibiotic, duration of prophylactic antibiotic, age, previous biopsy, the number of biopsy cores, number of patients, number of infectious complications, including fever and sepsis, were extracted. Subsequently, the risk ratio (RR) and 95% confidence intervals (CI) for infectious complications were retrieved. All discrepancies were resolved by consensus with coauthors.

### Quality assessment and risk of bias

Study quality and risk of bias were evaluated using the Risk-of-Bias version 2 (ROB) tool as outlined in the Cochrane Handbook for Systematic Reviews of Interventions [11]. The RoB assessment of each study was performed by two authors independently. We also performed the funnel plots.

### Statistical analysis

All statistical analyses were performed using R Version 4.2.2 (R Foundation for Statistical Computing, Vienna, Austria, 2023; meta). Statistical significance was set at *p* < 0.05. Forest plots with RR and 95% CI were calculated and depicted to assess the efficacy of PI disinfection before TRUS-PB. For further detailed investigation of infection complications, including fever and sepsis, subgroup analyses were conducted. Furthermore, we conducted subgroup analyses to clarify the comparative efficacy of PI with AP vs. AP alone. Cochrane’s *Q* tests and the *I*-square test were used to evaluate the heterogeneity. Significant heterogeneity was indicated by a *p* value < 0.05 in the Cochrane’s *Q* tests and *I*^2^ greater than 50%. When significant heterogeneity was observed, we attempted to investigate the causes of heterogeneity [12].

## Evidence synthesis

### Study selection and characteristics

**Fig. 1:**
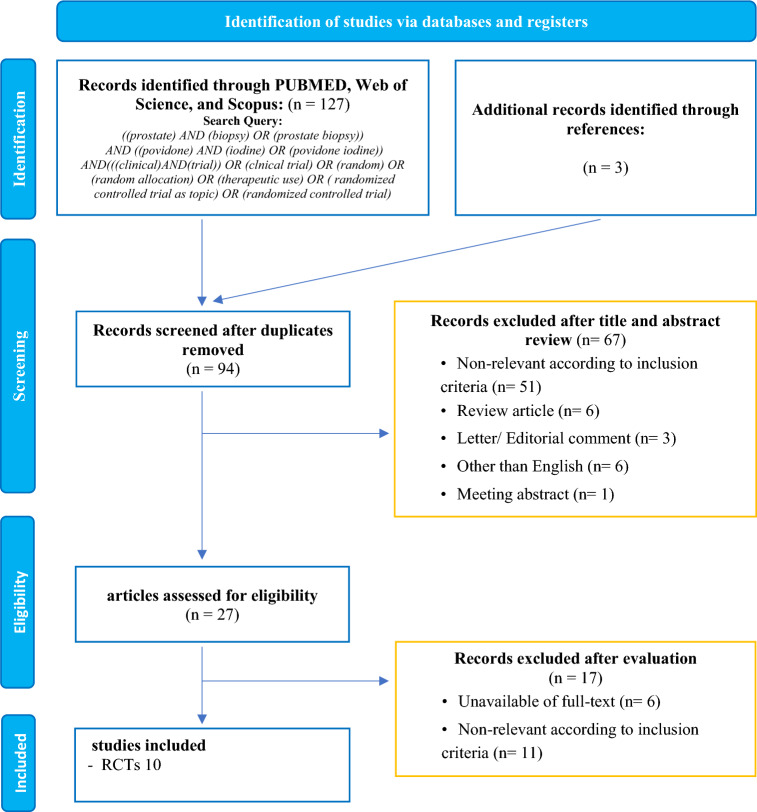
PRISMA flowchart

Our initial search identified 127 records of which 94 were screened for title and abstract after removing duplicates. Overall 27 articles were retrieved and 10 RCTs with 3,297 met our inclusion criteria (Fig. 1) [2, 5–8, 13–17]. All studies were prospective RCTs. The patient characteristics are summarized in Table 1. Of ten RCTs, nine studies used the AP. The types of APs included β-lactamase, fluoroquinolones, such as ciprofloxacin, levofloxacin, ofloxacin, and gentamicin. The detailed information on the usage and duration of these APs can be found in Table 1. Only one study, reported by Sharpe et al., did not use any AP before TRUS-PB [14]. All RCTs used fever as the primary outcome, and the recent six RCTs also used sepsis as a primary outcome [2, 5–8, 17]. Fever was defined differently across the RCTs, but it was characterized as body temperature higher than 37.8 ℃, 38 ℃ or 38.5 ℃. Sepsis was also defined differently across the studies. Some studies defined sepsis according to the Third International Consensus Definitions for Sepsis and Septic Shock [2, 18]. Other studies used the American College of Chest Physicians/Society of Critical Care Medicine Consensus Conference, which defined sepsis as having a positive urine or blood test along with at least two of the listed symptoms within a week of a biopsy. These symptoms include a temperature above 38.0 ℃ or below 36.0 ℃, a heart rate exceeding 90 bpm, breathing faster than 20 times a minute, a white blood cell count above 12.0 or below 4.0 × 10^9/L, or the presence of more than 10% immature forms [8, 19].

**Table 1:** Characteristics of included studies

Author	Published year	Nation	Design	No. of patients	Intervention	Control	AP	Primary outcome
Pontes-Junior	2022	Brazil	RCT	621	PI + AP	AP	Ciprofloxacin 500 mg PO/3 days	Fever, UTI, sepsis
Ergani	2019	Turkey	RCT	50	PI + AP	AP	Ciprofloxacin 500 mg PO + amikacin 1 g IM /3 days	UTI, sepsis
Ryu	2019	Korea	RCT	120	PI + AP	AP	Ceftriaxone 2 g IV	Fever, UTI, sepsis
Cadilhe	2017	Portugal	RCT	47	PI + AP	AP	Levofloxacin 500 mg PO/7 days	Fever, UTI, sepsis
Abughosh	2013	Canada	RCT	421	PI + AP	AP	Ciprofloxacin 1000 mg/3 days	Fever, UTI, sepsis
Ghafoori	2012	Iran	RCT	140	PI + AP	AP	Ofloxacin 300 mg/12 h + metronidazole 250/8 h/5 day	Fever, sepsis
Huang	2006	Taiwan	RCT	157	PI + AP	AP	Fluoroquinolone 1000 mg/day + ST were given twice daily for 3 days after the prostate biopsy	Fever
Melekos	1990	Greece	RCT	43	PI(+ AP)*	(AP)*	Piperacillin 2 g IV	Fever
Sharpe	1982	USA	RCT	40	PI	N/A	N/A	Fever
Brown	1981	USA	RCT	21	PI(+ AP)*	(AP)*	Gentamicin 80 mg IM	Fever

*AP* antibiotic prophylaxis, *PI* povidone-iodine, *RCT* randomized controlled trial, *ST* sulfamethoxazole plus trimethoprim *UTI*

^*^In these studies, not all patients used the antibiotic prophylaxis

### Risk of bias assessment

Authors’ judgments about each domain for each included study are graphed in Supplementary Fig. 1. Although some RCTs presented concerns in certain domains, two studies demonstrated low risk across all domains. Despite some observational studies showing low risk in specific categories, the majority of these studies displayed either moderate or serious overall risk of bias. Funnel plots of each analysis are depicted in Supplementary Fig. 1.

### Meta-analysis

#### Infectious complications (PI vs. non-PI)

**Fig. 2:**
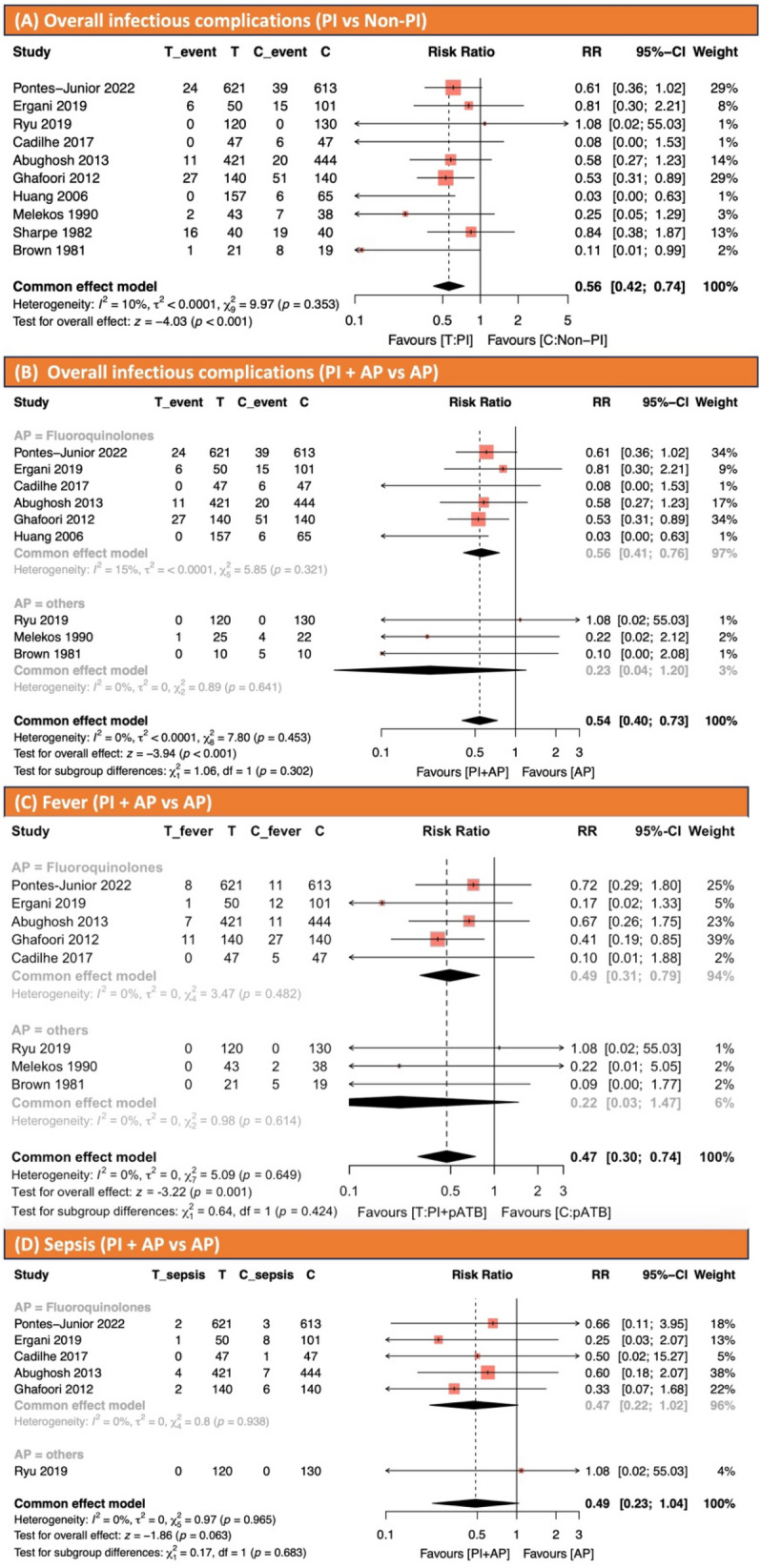
**A** Effect of povidone-iodine on infectious complications prior to transrectal ultrasound-guided prostate biopsy. **B** Effect of povidone-iodine and antibiotic prophylaxis on infectious complications prior to transrectal ultrasound-guided prostate biopsy. **C** Effect of povidone-iodine on fever prior to transrectal ultrasound-guided prostate biopsy. **D** Effect of povidone-iodine on sepsis prior to transrectal ultrasound-guided prostate biopsy. *AP* antibiotic prophylaxis, *PI* povidone-iodine

Ten studies have reported infectious complication in 3297 patients [2, 5–8, 13–17]. There were 1660 patients in the PI intervention group and 1637 patients in the non-PI control group. Eighty-seven patients (5.2%) in the intervention group and one hundred seventy-one patients (10.2%) in the control group experienced infectious complications. Nine studies reported infectious complications of fever, six studies reported sepsis, and five studies reported UTI. There was no heterogeneity. Infectious complication rates were significantly lower when the disinfection of rectal disinfection using PI was performed (RR 0.56, 95% CI 0.42–0.74; Fig. 2A).

##### Infectious complications (PI plus AP vs. AP monotherapy)

We conducted an analysis to further explore the efficacy of PI before TRUS-PB in an AP setting. Nine studies, comprising 3,163 patients, were analyzed for comparison overall infectious urinary complication rate between the PI plus AP group and AP monotherapy group [2, 5–8, 13, 15–17]. There were 1,592 patients in the PI plus AP group and 1572 patients in the AP monotherapy group. Sixty-nine patients (4.3%) in the intervention group and one hundred forty-six patients (9.3%) in the controlled group experienced infectious complications. The overall infectious complication rate was significantly lower in the PI plus AP group compared to the AP monotherapy group as well as PI compared to the non-PI (RR 0.54, 95% CI 0.40–0.73; Fig. 2B).

##### Fever (PI plus AP vs. AP monotherapy)

Then 8 studies, comprising 2941 patients, were analyzed for comparison between the PI plus AP group and the AP monotherapy group in terms of fever rate [2, 5–8, 13, 15, 17]. There were 1434 patients in the PI plus AP group and 1507 patients in the AP monotherapy group. Twenty-seven patients (1.8%) were in the intervention group and seventy-three patients (4.8%) in the controlled group experienced fever. The fever rates were significantly lower in the PI plus AP group compared to the AP monotherapy group (RR 0.47, 95% CI 0.30–0.74; Fig. 2C).

##### Sepsis (PI plus AP vs. AP monotherapy)

Six studies, comprising 2874 patients, were analyzed for comparison of sepsis rate between PI plus AP group and AP monotherapy group [2, 5–8, 17]. Five RCTs used ciprofloxacin, levofloxacin, or ofloxacin as AP [2, 5, 7, 8, 17]. Only one study, conducted by Rye et al., used ceftriaxone administered intravenously at a single dose of 2 g [6]. There were 1399 patients in the PI plus AP group and 1475 patients in the AP monotherapy group. Nine patients (0.64%) in the PI plus AP group and twenty-five patients (1.7%) in the controlled group experienced sepsis. There was no statistically significant difference between the PI plus AP and AP monotherapy group (RR 0.49, 95% CI 0.23–1.04; Fig. 2D).

## Discussion

We present the systematic review and meta-analyses that analyzed the effectiveness of rectal disinfection using PI prior to TRUS-PB. There are several key findings of our study. First, PI disinfection reduced the incidence rate of infectious complications in comparison to non-PI disinfection. Second, PI plus AP disinfection reduced the incidence rate of infectious complications in comparison to AP monotherapy. Third, we could not reveal the significant difference in sepsis rate between the PI plus AP group and the AP monotherapy group.

According to our analyses, PI plus AP was a significant reduction in the rate of fever (RR 0.47, 95% CI 0.30–0.74) and total infectious complications (RR 0.54, 95% CI 0.40–0.73) compared to AP monotherapy. This is in line with a previous study by Pu et al. [4] analyzing three RCTs and reporting the effectiveness of PI plus AP in reducing fever (RR 0.11, 95% CI 0.02–0.85), and total infectious complications (RR 0.23, 95% CI 0.10–0.54) compared to AP monotherapy. Based on the findings of previous studies and results of our present study, we confirmed the effectiveness of PI disinfection as prevention of infectious complications after TRUS-PB. Rectal cleansing with povidone-iodine has been shown to be safe and effective in reducing rectal flora counts [20]. Moreover, TRUS-PB transfer colonic bacteria into the prostate, risking infections like sepsis; hence, reducing bacterial translocation is crucial [21].

Our study indicated that disinfection using PI prior to TRUS-PB could not reduce the incidence rate of sepsis. However, these results should be interpreted with caution as the sepsis rate after TRUS-PB is generally very low; therefore, it is difficult to investigate whether there is a statistically significant effect of PI on reducing sepsis. We believe the incidence rate of sepsis (PI plus AP vs. AP: 0.6 and 1.6%) in our study should not be regarded as significant for both patients and urologists. Notably, among included studies, six RCTs [2, 5–8] did not reveal the efficacy of PI in reducing sepsis. Further studies are needed to clarify the efficacy of PI for preventing sepsis. However, it is clear that it is not possible to reduce the incidence rate of sepsis to zero with only PI disinfection. Thus, we believe the selection of an appropriate AP may be important. As previous guidelines traditionally recommended the use of fluoroquinolone, most of these RCTs used fluoroquinolone for AP. Recently, EAU guidelines have recommended against the use of fluoroquinolone for AP [10]. The European Commission has established rigorous regulatory requirements for fluoroquinolones use, leading to the withdrawal of recommendations for their use as peri-operative AP in procedures such as prostate biopsy [10, 22]. Our analysis also show no difference in overall infectious complications between the quinolone subgroup and other antibiotics, supporting this recommendation (*p* = 0.3). Instead of habitual fluoroquinolone usage, it is suggested that conducting rectal swab cultures prior to TRUS-PB may lead to a reduction in sepsis by allowing for the use of selective targeted AP [10].

Although we showed that intervention of PI plus AP before TRUS-PB could reduce infectious complications to 4.33%, we believe this percentage should not be considered negligible for either patients or urologists. Jim et al. revealed there was no infectious complication in TPUS-PB without AP group [23]. A comprehensive analysis of 165 studies encompassing 162,577 patients reported that the incidence of sepsis was 0.1% in cases of TPUS-PB and 0.8% in TRUS-PB [24]. Further studies are needed to investigate the best protocol to prevent infectious complication after TRUS-PB.

### Limitations

There are some limitations of our study. First, the duration and types of AP vary across ten included RCTs. No single study employed identical AP protocols. Moreover, the methods of iodine disinfection varied, including techniques such as rubbing with iodine-soaked gauze and rectal administration via syringe. In addition, the time before TRUS-PB and the waiting period after disinfection differed for each study. Second, there were some differences in the quality of bias among the RCTs. Thus, the results of our analysis have some limitations. Third, due to the low incidence of sepsis, there may not have been sufficient analysis. Finally, the patient-specific risk of infection, including diabetes, degree of urinary dysfunction, residual urine volume, and estimated prostate size, was unclear.

## Conclusion

Our analyses demonstrated that prophylaxis with PI prior to TRUS-PB reduced the risk of infectious complications compared with non-PI disinfection. We yield to detect a benefit to prophylaxis with PI prior to TRUS-PB in reducing the risk of sepsis compared with AP monotherapy. Although we did not detect a statistically significant difference in the rate of sepsis, the findings indicated a possible downward trend in its occurrence. Further research is needed to help develop a strategy to minimize the risk of infectious complications with prostate biopsy.

## Supplementary Information

Below is the link to the electronic supplementary material.

Supplementary file1 (DOCX 6111 KB)

## Data Availability

Data can be provided upon inquiry.
